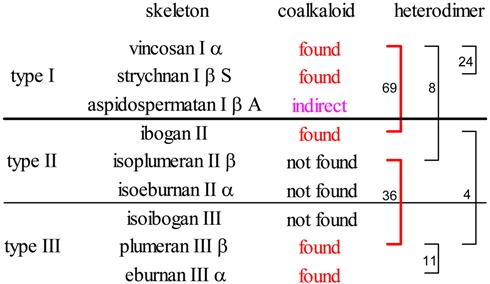# Rigorous Biogenetic Network for a Group of Indole Alkaloids Derived from Strictosidine^†^

**DOI:** 10.3390/molecules13081875

**Published:** 2008-08-27

**Authors:** László F. Szabó

**Affiliations:** Department of Organic Chemistry, Semmelweis University, Hőgyes utca 7, H-1092 Budapest, Hungary; E-mail: szalasz@szerves.sote.hu

**Keywords:** Secologanin, coalkaloid, dimer alkaloid, reaction mechanism, computer searching, chemotaxonomy

## Abstract

Strictosidine, the precursor of more than 2,500 indole alkaloids, was isolated from four species of three plant families. By searching the Dictionary of Natural Products on DVD it was found that about 150 indole alkaloids were obtained from the same species (coalkaloids), which is a direct proof of their common origin. On the base of their three-dimensional structure, taxonomic properties and standard reaction mechanisms an extended network was established which involved the four fundamental skeletons, the three types of carbon framework in the secologanin subunit and all major groups of indole alkaloids derived from secologanin and tryptamine (except a few minor groups, in which only less then 10 alkaloids were known). The system was extended to the heterodimer indole alkaloids and the quinoindole alkaloids as well.

## Introduction

The monoterpenoid indole alkaloids form a uniform group of natural products. Several generations of researchers have worked on their isolation, structure determination, chemical transformations and synthesis. Much effort has also gone into elucidating their biogenesis, and as a consequence, the main pathways of their formation are already known [1,2,3,4]. However, the applied methods consume much time, material and human energy. In addition, it is often dubious if the postulated *in vitro* or biomimetic reactions really take place *in vivo* [5]. There is a certain gap between the level of the biological micromolecules (alkaloids) and macromolecules (enzymes and genes), and it is difficult to follow the processes on the micromolecular level. Although a large amount of knowledge has been accumulated concerning the starting steps and the formation of some key compounds [6,7,8], many questions are still unanswered at the more remote points and in special groups.

Recently, specialized data bases and their electronic searching features have offered an alternative tool for chemotaxonomic studies. The fact that more than 2,500 indole alkaloids were isolated mainly from three plant families (Rubiaceae/Naucleaceae RUB, Loganiaceae/Strychnaceae LOG and Apocynaceae APO) and formed from two building blocks [secologanin (**1)** and tryptamine (**2**) or tryptophane] through a single precursor [strictosidine (**3**)] suggested a strong coherence in this collection of alkaloids. Compounds isolated from the same cells, species, genera and/or families in parallel and/or consecutive reactions may be guideposts in their genesis, and in cases where highly reactive or sensitive intermediates cannot be isolated in intact form from biological sources, they can be postulated according to standard organic reaction mechanisms. The rich collections of structural, physical, chemical and taxonomic data effectively contribute to such an approach. It seems, therefore, that the isolated compounds themselves could give a more realistic basis and complementary method for these studies.

Our previous work in the chemistry of secologanine and especially of strictosidine provided good starting points [9,10]. The internal consistance was further supported by the mechanism of chirality transfer at C-15 and C-3 in the type I class of indole alkaloids, and some stereoselectivity could be demonstrated even in the type II and III classes as well [11,12]. Extended biogenetic networks could be established by studies in the large group of type I alkaloids [13] and in the small but highly specific group of *Melodinus* alkaloids [14]. In addition, a long evolutionary line was constructed for the formation of ellipticine from ajmalicine (corynanthean skeleton) and an analogous line postulated for olivacine from isodihydrocadambine (malindan skeleton) [15].

In this paper we look for those indole alkaloids which have a direct chemotaxonomic contact with strictosidine, the primary precursor of indole alkaloids. In this relation two main questions were to be answered:

1. Compounds isolated from the same species reveal their common origin. Which alkaloids could be isolated from those plant species which produce also strictosidine (**3**)? In other terms: which are the “coalkaloids” of this universal precursor of indole alkaloids?
2. The monomer components necessarily coexist in the same species, from which the dimer alkaloids were isolated. This fact likewise reveals chemotaxonomic connections among the individual alkaloids and species. Which types of monomer components are connected in the dimers?

As the reaction mechnisms were already discussed previously or will be so in the future, they are mentioned in this paper only briefly and schematically, however, the chemotaxonomic connections are analyzed in detail.

**Scheme 1:**
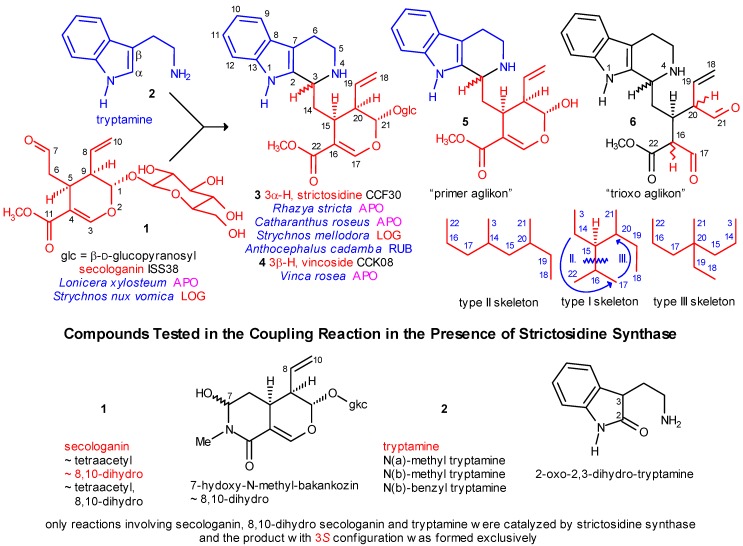
The Coupling Reaction.

The necessary structural, chemical and taxonomic data were taken from the database Dictionary of Natural Products on DVD Version 16.2 (in the following DNP) [16], and completed occasionally with data from the Beilstein Crossfire and the Chemical Abstracts online data bases. In the structural formulas and throughout the text, the biogenetic numbering system shown in formula of strictosidine (**3**) was used [17]. The only exceptions are the building blocks **1** and **2**, which are numbered as indicated in their own formulas. All isolated compounds in the schemes and in Table 1 were provided with Chapman and Hall (C&H) code numbers, which give access to the rich primary literature sources. The figures giving the numbers of alkaloids in the different taxons are approximative and in several cases only estimations, as they are changed by the continuous renewal of the database and depend on the selection principles used as well.

## The Biogenetic-type System of Indole Alkaloids

In this work the indole alkaloids natural products are considered, which are formed from secologanine (**1**) and tryptamine (**2**) (or tryptophan). However, even within this field certain limitations had to be applied.

**Scheme 2:**
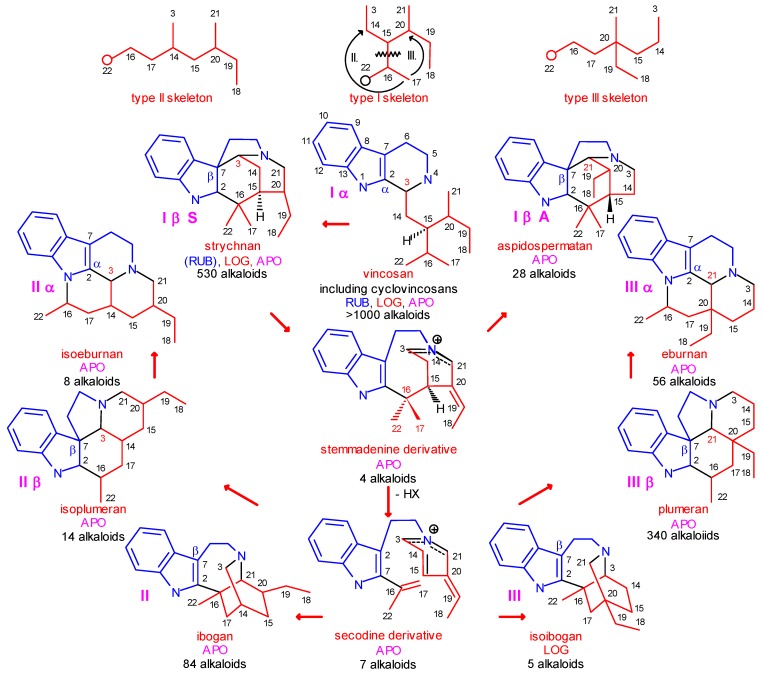
System of Indole Alkaloids Derived from Secologanin.

In the “quinoindole” alkaloids group (about 100 isolated natural products), the original indole subunit is extended into a quinoline subunit during the biosynthesis. They are dispersed among all three plant families mentioned above, as well as in some additional ones. Their special groups are the camptothecan (in the Icacinaceae and Nyssaceae families), as well as the cinchonan and melodan alkaloids. In another group of alkaloids derived from secologanin, the heterocyclic base in the coupling reaction is a substituted phenylethyl amine and provides the isoquinoline ring system which was found in some 90 isolated natural products (*“Ipecacuanha”* alkaloids) mainly in the Rubiaceae and Alangiaceae families. The problem of the quinoindole alkaloids will be discussed briefly, and the group of the *Ipecacuanha* alkaloids temporarily disregarded.

Previously, the system of indole alkaloids was based mainly on formal, structural properties of the individual compounds [18,19]. For our purposes, a chemically *and* biologically oriented system was constructed [11] which is shown in Scheme 2 with slight modifications. It is based on the following principles:

level 0: formation of strictosidine (**3**) from secologanin and tryptamine and removal of the glucosyl subunit (Scheme 1);
level 1: cyclization of the secologanin subunit to N-1 or/and N-4 of the tryptamine subunit (Scheme 2 shows only cyclizations to N-4);
level 2: in the secologanin subunit, transformation of the type I skeleton into the type II and type III ones (Scheme 1);
level 3: attachment of C-3 or C-21 or neither of them to C-2 (α) or C-7 (β) position of the the indole ring;
level 4: further cyclizations between the secologanin and the tryptamine subunits;
level 5: further cyclizations inside the secologanin subunit;
level 6: further transformations (e.g. rearrangements, ring extensions and ring contractions; fragmentation of the tryptamine side chain, formation of sesqui- and dimers, which are temporarily omitted).

The system is shown in Scheme 2 up to level 4. It has an internal consistancy and predictive power, hard in principles, but soft for accomodating any existing or new alkaloid.

## The Main Precursor Strictosidine

The precursor of most indole alkaloids is strictosidine (**3**), having *S* configuration at C-3 (Scheme 1). Its formation from secologanin and tryptamine is catalyzed by the enzyme strictosidine synthase. Both the chemo- and stereoselectivities of the enzyme are high *in vitro* (i.e. under cell-free conditions). It was demonstrated [20], that of the components shown in Scheme 1 only the 8,10-dihydro derivative of **1** (of course over **1** and **2**) can participate in the coupling reaction, and no trace of vincoside (**4**) (with 3*R* configuration) was formed. In the absence of the enzyme the reaction is much slower, and neither chemically, nor stereochemically selective [9a]. The complete stereoselectivity is remarkable, because strictosidine synthase was isolated both from *Catharanthus roseus* [21] and *Rauwolfia serpentina* [22]. From the first species strictosidine (**3**) with 3*S* and vincoside (**4**) with 3*R* configuration were isolated, and from the second species ajmaline (GQZ67) with 3*S* and reserpine (CFD56) with 3*R* configuration. This raises the obvious question, what is the source of the 3*R* chirality in the species mentioned above? The answer is not known yet.

The *S* chirality of C-15 in both compounds comes from the C-5 center of secologanin. Most of the type I alkaloids having a bridged skeleton with participation of C-15 (e.g. the akuammidan, akuammilan, pleiocarpaman, strychnan and aspidospermatan alkaloids) have *S* configuration at C-3, which suggests that their precursor is strictosidine, and the formation of the bridge is possible only with the *cis* orientation of the hydrogens attached to C-3 and C-15 [11a]. However, in a limited number of alkaloids with fused skeleta, the configuration at C-3 is *R*. The cause of it is likewise unknown.

**Scheme 3:**
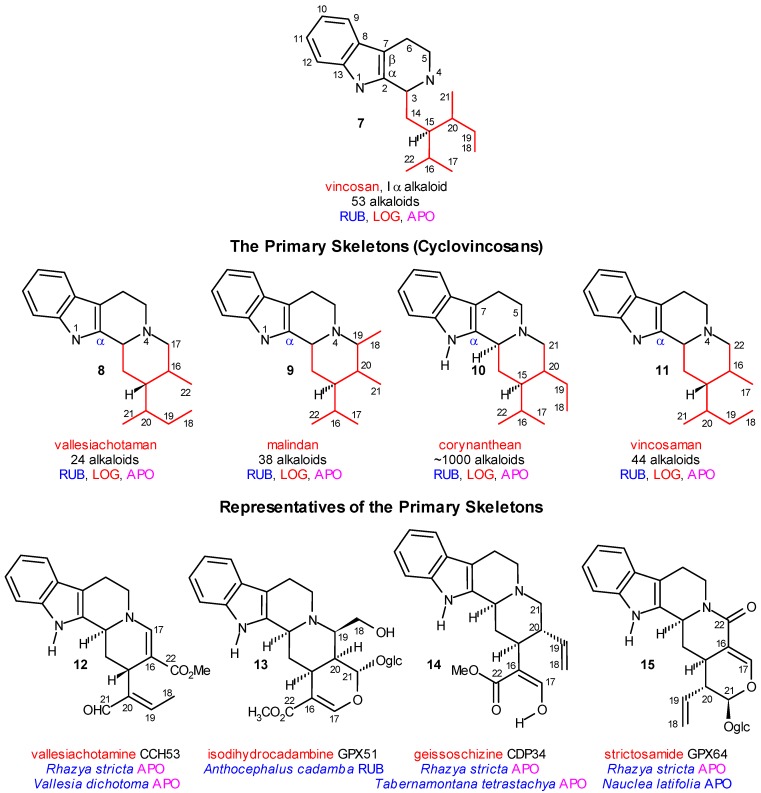
Primary Cyclizations of Vincosan.

Strictosidine (like vincoside) belongs to the type I α alkaloids, i.e. it contains the secologanin subunit in its unrearranged form, and its C-3 is attached to the α-position of the indole ring (vincosan skeleton **7** in Scheme 3). The biosynthetic power of strictosidine is mainly revealed after deglucosylation. The primarily formed aglucone **5** cannot be isolated, as it undergoes many types of isomerizations (which are represented in the trioxo form **6** in Scheme 1) [23]. Subsequent cyclizations and further transformations provide a high number of type I α alkaloids, whose formation was discussed previously [13].

Strictosidine was isolated from four plant species (in this paper they are called “strictogenic species”) ranked into three plant families (*Anthocephalus cadamba*, Rubiaceae, *Strychnos mellodora*, Loganiaceae, *Catharanthus roseus* (=*Vinca rosea*; the two names are considered as synonyms, and in this paper mainly the first name is used) and *Rhazya stricta*, Apocynaceae). The next step in the further molecular evolution of strictosidine is the primary cyclization between the nucleophilic N-4 center and one of the four electrophilic centers (C-17, C-19, C-21 and C-22) of the secologanin subunit, which gives the vallesiachotaman, malindan, corynanthean and vincosaman skeletons **8**-**11**, respectively (primary “azacyclizations”). These “cyclovincosan” skeletons and their probable first representatives (**12**, **13**, **14**, and **15**) are shown in Scheme 3. During the biosynthesis, some structural elements (mainly C-17 or C-22) may be lost (“truncated” alkaloids). Analogous cyclizations also take place with participation of N-1, and gives nearly 100 alkaloids, however, they will not be discussed in this paper.

## Coalkaloids of Strictosidine in Type I α Class

**Scheme 4:**
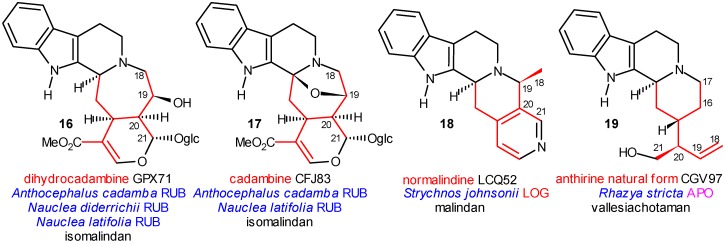
Further Representatives of the Malindan and Vallesiachotaman Skeletons.

Fortunately, coalkaloids resulting from all four types of azacyclizations were found in the strictogenic species, as is shown in Scheme 3 and Scheme 4, as well as in Table 1. Azacyclization to C-22 corresponds to a simple (often spontaneous) lactamization. The group of the vincosaman alkaloids, **11**, is rather homogenous, and contains nearly 50 alkaloids, among others strictosamide (**15**) from the strictogenic *Strychnos mellodora* (Loganiaceae), and its C-3 epimer vincosamide from *Adina rubescens* (Rubiaceae), found as glucosides.

Azacyclization to C-19 (Scheme 4) results in the formation of the malindan skeleton **9**. It can take place likewise on the glycosidic level, after activation of C-19 of the vinyl group, e.g. by epoxidation. This activation is known in secologanin chemistry [24], and is suggested in the present case by the hydroxy group at C-18 of isodihydrocadambine (**13**). The corresponding glycoside was isolated from the strictogenic *Anthocephalus cadamba*, Rubiaceae. The six-membered ring D of the fused cyclic system might be extended into a seven-membered azepin ring (20(19→18)-abeo-) (isomalindan skeleton), which was supported by isolation of dihydrocadambine (**16**) and cadambine (**17**) from the same species (Scheme 4). The (iso)malindan skeleton appears in more than 40 alkaloids (e.g. normalindine, **18**), partially also on higher levels and even at the end of the molecular evolution, in the derivatives of olivacine (**66**) (Scheme 12).

**Table 1:** Coalkaloids of Strictosidine (parent name, number of derivatives, C&H number, type of skeleton).

*Anthocephalus cadamba* (RUB) (8 alkaloids)	*Rhazya stricta* (APO) (50 alkaloids)
Cadambine, CFJ 83, isomalindan	Akuammicine deriv. GNP98, strychnan
Cadamine, 2 derivs. HFP34, GZM28, malindan	Akuammidine CGS53, akuammidan
Dihydrocadambine, 2 derivs. GPX71, GPX73, isomalindan	Akuammiline deriv. CGS78, akuammilan
Isodihydrocadambine, 2 derivs. GPX51, GPX53, malindan	Anthirine deriv. CGV97, anthiran
**Strictosidine, CCF30, vincosan**	Aspidospermidine 2 derivs. NSG56, NGX40, plumeran
	Bharhingine GRD15, strychnan
*Catharanthus roseus* (= *Vinca Rosea*) (APO) (49 alkaloids)	Burnamine deriv. CGP15, akuammilan
Ajmalicine deriv. BFV84 (VR), GNX93, corynanthean	Decarboxymethoxytetrahydrosecodine deriv NQD29 secodan
Ajmalicine hydroxyindolenine deriv. GNX49, corynanthean	Dihydrocorynantheol deriv. GNZ33, corynanthean
Akuammicine deriv. CGS34, strychnan	Dihydroeburnamenine LCR84, eburnan
Akuammiline deriv. LTT85, akuammilan	Eburenine 2 derivs. GZP25, LNJ42, plumeran
Alioline, OSQ04, ibogan	Geissoschizine CDP34, corynanthean
Alstonine 2 derivs. GNX52 (VR), CCC44, corynanthean	Isorhazicine BQR50, secoajmalan
Apparicine (S)-form, CGW23, vallesaman	Isositsirikine 4 derivs. LCS06, CCW87, NBV19, NBV18, corynanthean
Bannucine CQG04, plumeran	Lanceomigine deriv. CHL97, akuammilan
Catharanthine CFM58 ibogan (VR)	Leuconolam NNZ31, plumeran
Fluorocarpamine indoxyl deriv. GRG17 pleiocarpaman	Nor-C-fluorocurarine deriv. HHZ45, strychnan
Isositsirikine 3 derivs. CCW78 (VR), CCW86, CCW87, corynanthean	Quebrachamine deriv. CFB72, plumeran
Lochneridine BQS00 (VR) strychnan	Rhazidigenine hydroxyindolenine 2 derivs. CFF52, CFF54, plumeran
Perivine 2 derivs. CGN81 (VR), CGN86 (VR), vobasan	Rhazimine 2 (quinoline) derivs. CFK02, CFK05, FYL18, akuammilan
Preakuammicine CGQ70 (VR), strychnan	Rhazinaline 2 derivs. CDR88, NXF68, akuammilan
Rosicine CFK07, plumeran/isoplumeran	Rhazinilam 2 derivs. GPF00, FNO12, plumeran
Sarpagin deriv BCC19 (VR) akuammidan	Rhazizine LDC76, strychnan
Sitsirikine 2 derivs. HJQ31 (VR), BCF50 (VR), corynanthean	Secodine 2 derivs. CHM24, BFV95, secodan
Strictamine deriv. CFF61, akuammilan	Stemmadenine CCD41, strychnan/aspidospermatan
**Strictosidine CCF30 (+VR), vincosan**	Strictamine 3 derivs. HHT53, CDR79, BFY65, akuammilan
Strictosidine 2 derivs. CCK08 (VR), CCK10 (VR)	Strictanine BQS42, plumeran
Tabersonine 2 derivs. GQZ42, HJQ42 (VR), plumeran	Stricticine GRD24, GRG17, plumeran
Talpinine deriv. GQW95, akuammidan	Strictine GRD25, pleiocarpaman
Tombozine deriv. GRC35 (+VR), akuammidan	Strictosamide GPX64, vincosaman
Venalstonine deriv. CCH72, plumeran	**Strictosidine CCF30, vincosan**
Vincadifformine deriv. CCJ46 (VR), plumeran	Vallesiachotamine 3 derivs. CCH54, CCH55, LXQ87, anthiran
Vincarodine GNK80, eburnan	Vincadifformine 3 derivs. HBN54, LHX61, CCJ49, plumeran
Vincoline CCK01 (VR), plumeran	Vincamajine deriv. LJB72, akuammidan
Vindolidine 5 derivs. KLJ88 (+VR), JHQ68, JRJ50 GQZ38 GQZ39, plumeran	
Vindolinine 4 derivs. BQX53 (+VR), MVY20, BQX56, BQX57, plumeran	*Strychnos mellodora* (LOG) (5 alkaloids)
Voalutein indoxyl deriv. CFK06, ibogan	Lyaloside 2 derivs. GVY09, GVY10, vincosan
(VR) indicates alkaloids isolated (also) from Vinca rosea	**Strictosidine CCF30, vincosan**
	Strictosidine 2 derivs. LHX56, MTN39, vincosan

After deglucosylation, the other two previously masked electrophilic centers will be free, and the azacyclizations involving C-17 and C-21, respectively, can take place. Azacyclization to C-17 provides the vallesiachotaman skeleton **8**, with nearly 25 compounds, in the majority of which C-22 is lost during the biosynthesis (i.e. .in the anthirine derivatives). Both vallesiachotamine (**12**) and anthirine (**19**), the characteristic alkaloids of this group, were isolated from the strictogenic *Catharanthus roseus* (Apocynaceae). If C-17 (in vallesiachotaman) or C-22 (in vincosaman) was removed during the biogenetic process, the two “truncated” skeletons **8** and **11** became formally (but not biogenetically) indentical.

**Scheme 5:**
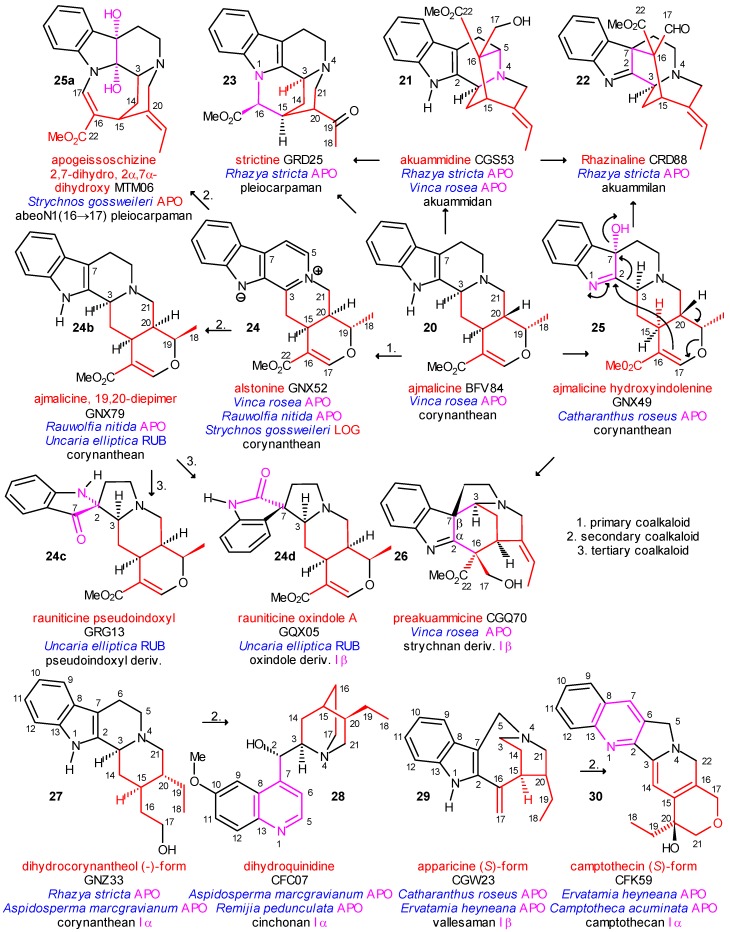
Further Cyclic Systems Derived from the Corynanthean Skeleton.

N-4 azacyclization to C-21 gives the corynanthean skeleton **10**, which was found in nearly a thousand compounds, and provides the largest subclass of indole alkaloids. One of its first representatives is geissoschizine (**14**). Some additional characteristic structures are shown in Scheme 5. There are two main sources of the great variability of the corynanthean alkaloids: “secondary” cyclizations and oxidations.

The central element of the additional cyclizations is *C*-16 and mainly directed to C-5, C-7 or N-1. In the first case the akuammidan (including also the ajmalan skeleton), in the second the akuammilan, and in the third the pleicarpaman skeletons are formed. They are represented in Scheme 5 by akuammidine (**21**), rhazinaline (**22**) and strictine (**23**), respectively; all were isolated from the strictogenic *Rhazya stricta* and are primary coalkaloids of strictosidine. A typical compound in this evolutionary line is ajmalicine (**20**), isolated from the likewise strictogenic *Vinca rosea*. From this species several other ajmalicine derivatives were obtained. One of them is alstonine (**24**), a stereoisomer of ajmalicine (**20**), in which, however, ring C is aromatized. Fortunately, **24** was also isolated from *Rauwolfia nitida* and *Strychnos gossweileri*, and according to this fact, it may be a biogenetic link in the pathway towards the oxidized derivatives of the corynanthean skeleton.

From *Strychnos gossweileri* a dihydroxy derivative **24a** of the unnatural apogeissoschizine could be obtained, which is a secondary coalkaloid of strictosidine. Its skeleton may be considered a 1(16→17)-abeo-derivative of the pleiocarpaman skeleton in **23**. In *Rauwolfia nitida* a further ajmalicine stereoisomer **24b**, likewise a secondary coalkaloid, was found, which was isolated from *Uncaria elliptica* as well. This last species, which belongs already to the Rubiaceae family, also provided the two main representatives of the pseudoindoxyl and the oxindole alkaloids, rauniticine pseudoindoxyl (**24c**) and rauniticine oxindole A (**24d**), respectively, both of which are tertiary coalkaloids. As it is shown, there are grades in the coalkaloid relation depending on the number of species concerning the parent and the derived compounds in each case. when the name “coalkaloid” is used without qualification (secondary, etc.), they are considered as primary coalkaloids, i. e. isolated from one common species.

Finally, ajmalicine hydroxyindolenine (**25**), a fourth variant of the oxidized I α alkaloids was isolated from the tissue culture of strictogenic *Catharanthus roseus*. Its main importance is shown in its formula: according to the electron stream indicated by curved arrows in the formula of **25**, C-3 can be shifted from C-2 (indole α-position) to C-7 (indole β-position) and simultaneously (or subsequently) C-16 attached to C-2, i.e. a type I α alkaloid could be transformed into a type I β alkaloid. After a potential reduction of the formyl group, the first representative of the strychnan alkaloids, preakuammicine (**26**) would be formed similarly in *Catharanthus roseus*. It is clear that already the type I oxindole alkaloids should be considered as type I β compounds because C-3 is attached to C-7 (β-position of the indole ring) in them. It is also known that the oxindole, pseudoindoxyl and hydroxyindolenine derivatives were found in the type II and type III alkaloids as well. The oxidative processes shown above were carried out also under *in vitro* conditions [25,26,27]. The chemical mechanisms of the cyclizations and the formation of the oxidative derivatives were discussed previously [13a]. In addition, the last line of Scheme 5 shows that this analysis could even be extended to the two important subgroups of the “quinoindole alkaloids”. Both dihydrocorynantheol (-)-form **27** and apparicine (*S*)-form **29** were isolated from strictogenic species (primary coalkaloids from *Rhazya stricta* and *Catharanthus roseus*, respectively). The first, **27**, was obtained also from *Aspidosperma marcgravianum* which provided also dihydroquinidine (**28**, cinchonan skeleton), the other, **29**, was found also in *Ervatamia heyneana*, together with camptothecine (*S*)-form (**30**, camptothecan skeleton), consequently both **28** and **30** are secondary coalkaloids of strictosidine.

Of course, the coexistance of these alkaloids (in Scheme 5 and elsewhere) in the given species does not mean that they are formed immediately one from other, but more probably from some common precursors which, however, were not found in natural sources, perhaps because of their high reactivity. As a final conclusion, it should be remarked, that of the four primary azacyclizations only the malindan and the corynanthean skeletons may be transformed into the type I β structure.

## Coalkaloids of Strictosidine in Type I β Class

**Scheme 6:**
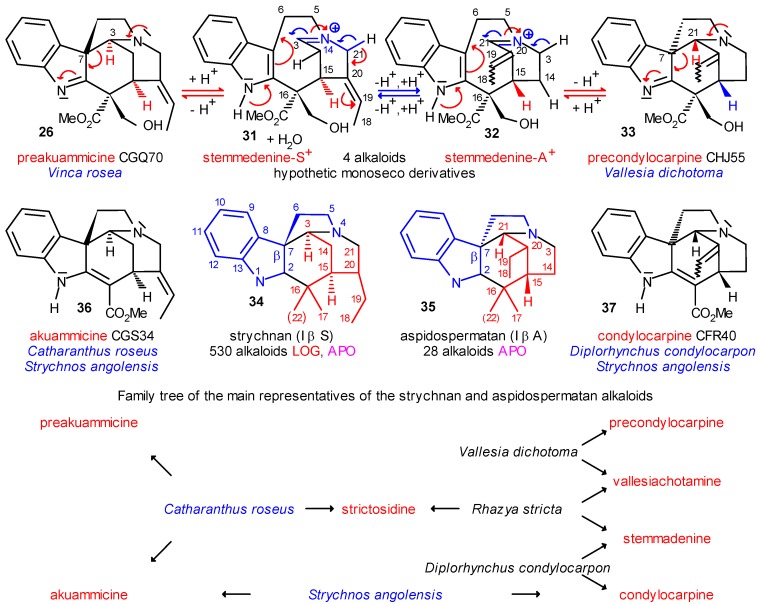
Chemotaxonomic Interrelations between Strychnan and Aspidospermatan Alkaloids.

The three main subclasses of the type I β alkaloids are the type I oxindoles (e. g. **24d**, Scheme 5), the strychnan (I β S) (**34**) and the aspidospermatan (I β A) (**35**) alkaloids (Scheme 6). The strychnan group **34** is rather uniform and its alkaloids (together with the type I oxindole alkaloids) number nearly 400. Several coalkaloids of strictosidine were found among them. The small group (less than 30 compounds) of the aspidospermatan alkaloids **35** is likewise uniform, but has no coalkaloid of strictosidine **3** and only indirect chemotaxonomic connections to it. Therefore it is of high importance that the formation of the aspidospermatan alkaloids (I β A) is biogenetically strongly related to that of the strychnan alkaloids (I β S), as is shown in the formulas and family tree of Scheme 6.

In the formula of preakuammicine (**26**) curved arrows show the electron stream in the N-4→N-1 direction, which is increased by protonation. The result may be the cleavage of the C-3–C-7 bond and the formation of the charged monoseco structure stemmadenine-S^+^ (**31**), which can be tautomerized by deprotonation–reprotonation to give stemmadenine-A^+^ (**32**). In these intermediates the tryptamine and secologanin subunits can be turned around one another along the C-5–C-6 bond and around C-16, consequently in a further proton-catalyzed step, the C-7 center can be reclosed either to the original C-3 in **31** or to the alternative C-21 center in **32**, as the centers are in analogous positions. In this latter case, after deprotonation, precondylocarpine **33** (aspidospermatan skeleton) can be formed. Preakuammicine (**26**) and precondylocarpine (**33**) are structural isomers formed by simple rotation and without any change of chirality at C-15. Although the charged stemmadenine-S^+^ (**31**) and stemmadenine-A^+^ (**32**) are hypothetic monoseco intermediates, some of their reduced derivatives (e. g. stemmadenine, **38**) were found in the strictogenic *Rhazya stricta* (and other species as well).

There is a tight chemotaxonomic connection between these structural and stereoisomers. Preakuammicine (**26**) from *Catharanthus roseus* is a primary coalkaloid of strictosidine, unlike precondylocarpin (**33**), which could be isolated only from the non-strictogenic *Vallesia dichotoma*. However, from this species vallesiachotamine was likewise isolated which could be obtained from *Rhazya stricta* as well. The chemotaxonomic relation is further supported in the case of the analogous pair akuammicine (**36**, strychnan) and condylocarpine (**37**, aspidospermatan), which are in the same structural and stereoisomeric relation as their precursors **26** and **33**, and both were isolated from *Strychnos angolensis*. Akuammicine (**36**) was directly obtained also from the strictogenic *Catharanthus roseus*. On the other line the contact is indirect: condylocarpine (**37**) was isolated from *Diplorhynchus condylocarpon* producing also stemmadenine (**38**), which was isolated from *Rhazya stricta* (and from several other species as well). In the family tree it can easily be recognized that preakuammicine and akuammicine are primary, and precondylocarpine and condylocarpin secondary coalkaloids of strictosidine; in addition, preakuammicine and akuammicine, as well as akuammicine and condylocarpine, are primary, while precondylocarpine and condylocarpine are tertiary coalkaloids of each other.

Tautomeric stemmadenine-S^+^ (**31**) and stemmadenine-A^+^ (**32**) may be starting points in the formation of those indole alkaloids in which the ethylene side chain of the tryptamine subunit is partially or completely absent (vallesaman, ulean, ellipticen and olivacen skeletons). In these groups until now only a single coalkaloid, apparicine (**29**, Scheme 5) was found, however, by detailed analysis some indirect chemotaxonomic relations could be demonstrated. Moreover, of the azacyclized skeletons **8**–**11** only the malindan and the corynanthean skeletons **9** and **11** can undergo this degradation, as is proved by isolation of both olivacine (**66**) and ellipticine (**65**), respectively (Scheme 12). But details of this work are already beyond the limits of the present paper [13b].

## The Central Role of Stemmadenine and Secodine Derivatives

**Scheme 7:**
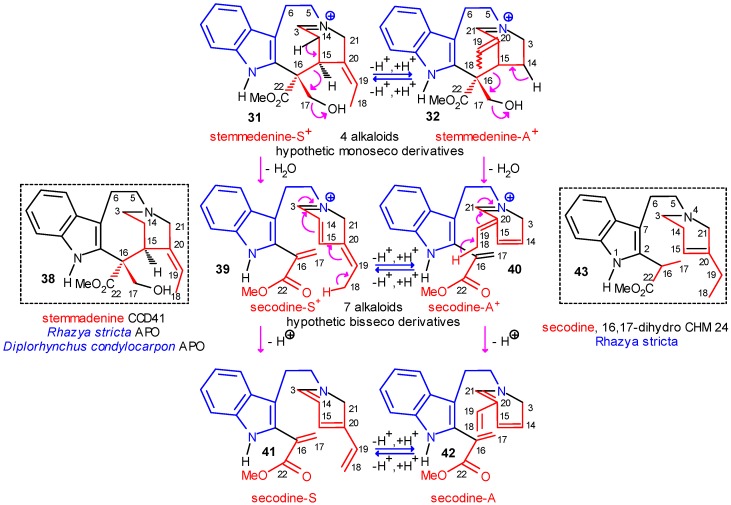
Central Role of Stemmadenine and Secodine Derivatives.

**Scheme 8:**
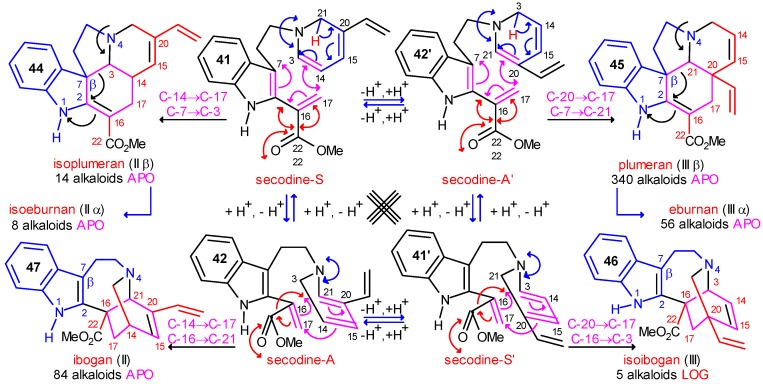
Formation of Type II and Type III Alkaloids.

Now, the molecular evolution of the indole alkaloids is arrived at an important phase which is shown in Scheme 7 and Scheme 8. In the charged tautomeric structures **31** and **32** shown in Scheme 7, ocurrence of a further fragmentation (shown again by curved arrows) is possible, which probably requires activation at C-17 either by enzyme or by acetylation. The possible fragmentation would involve the cleavage of bond C-15–C-16 (“strategic bond”) by which the bisseco tautomeric and charged structures secodine-S^+^ (**39**) and secodine-A^+^ (**40**) could be formed. This bond can really be cleaved under mild abiotic conditions by proton or base catalysis in simple vincoside derivatives [9b] or even in secologanin derivatives as well [28]. In the charged structures **39** and **40** extended conjugated systems are present, from which neutral tautomeric secodine-S (**41**) and secodine-A (**42**) can be formed. Though all these structures are again hypothetic intermediates, some alkaloids stabilized by hydrogenation were isolated from natural sources [e.g. 16,17-dihydrosecodine (**43**) from *Rhazya stricta*].

In the structures of neutral conformer pairs secodine-S **41=41’** and secodine-A **42=42’** (Scheme 8) strongly polarized conjugated double bond systems are preformed, which may formally undergo four types of Diels–Alder reactions, as indicated by curved arrows in Scheme 8. In these cyclizations the isoplumeran (type II β), plumeran (type III β), isoibogan (type III) and ibogan (type II) skeletons **44**-**47** can be formed. The details of these possible transformations were previously analyzed [11b]. Here it should be noted only that in some alkaloids of the new, rearranged type II and type III skeletons the double bonds (indicated by black lines) are conserved or formed just in the positions which are required by the hypothetical Diels–Alder reactions. It should also be mentioned that in the synthetic work by G. Kalaus, C. Szántay *et al.* for preparation of pandoline-like molecules, detailed quantum chemical calculations supported the two-step character of an analogous transformation [29].

In alkaloids having the isoplumeran (II β) or plumeran (III β) skeletons **44** and **45**, C-7 (β position in the indole ring) has a covalent bond to C-3 or C-21, respectively. This bond can be shifted to the 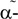
position of the indole ring (arrows in the formulas) and give in several steps the eburnan (III α) and isoeburnan (II α) alkaloids (see in Scheme 2), respectively. This would be the last phase in the molecular evolution of the indole alkaloids. Finally, it should be emphasized that the transition from the type I to the type II/III class of indole alkaloids is possible only in the evolution line of the corynanthean skeleton.

## Coalkaloids of Strictosidine in Type II and Type III Classes

In alkaloids which are formed after rearrangement of the original (type I) carbon skeleton of secologanin subunit into the type II and type III ones, the number of coalkaloids is strongly reduced, however several of them were found. The isolation of natural products from their biological sources rarely means a fully systematic work; many different and accidental motivations influence which species of which families were investigated and which compounds obtained from them. Therefore the number of isolated compounds is very different.

**Scheme 9:**
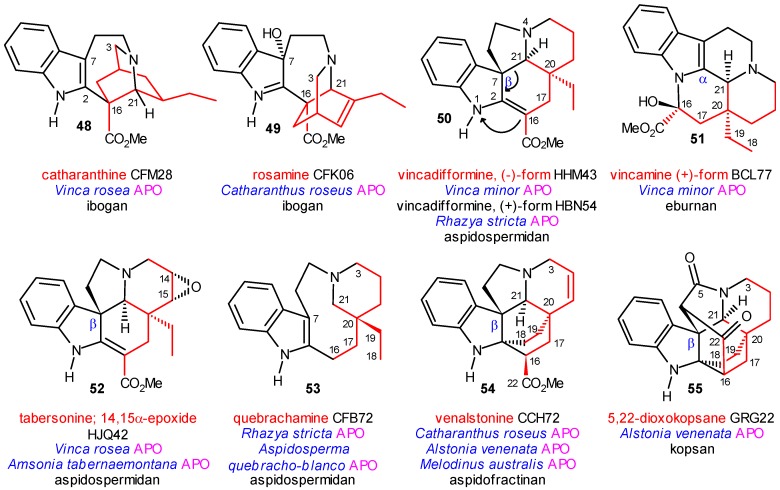
Some Coalkaloids with Rearranged Secologanin Subunits.

The plumeran group with its nearly 400 individual compounds represents the third largest group of indole alkaloids, the isoibogan and isoeburnan groups are the smallest ones with 5 and 8 natural products, respectively. In these latter two groups, as well as in the isoplumeran group no coalkaloids of strictosidine were found. Some representatives of really existing coalkaloids having a rearranged secologanin subunit are shown in Scheme 9.

The ibogan group shows an interesting phenomenon: in the secologanin subunit of catharanthine (**48**) all chirality centers have configurations opposite to those found in rosamine (**49**) (and other ibogan alkaloids), however, both were isolated from strictogenic species. The aspidospermidan subgroup of the plumeran alkaloids has several monomer alkaloids isolated from strictogenic species (e.g. **50**, **52** and **53**), and in addition dimeric alkaloids of vinblastine (**57**, Scheme 11) as well.

Interestingly, both enantiomers of vincadifformine (**50**) were isolated from plants: the (+)-form from the strictogenic *Rhazya stricta*, the (‑) form from the closely related *Vinca minor*. From the same species the eburnan alkaloid vincamine (**51**) was also obtained.

The transformation of vincadifformine into vincamine is indicated by curved arrows in formula **50** and was carried out in several steps even under abiotic conditions [30]. In the tabersonine derivative **52** the position of the epoxide oxygen shows the place of the previous double bond, which remained after the hypothetic Diels–Alder cyclization. In the aspidofractinan subgroup the sole alkaloid is venalstonine (**54**), which was obtained from the strictogenic *Catharanthus roseus*. However, it is a key structure isolated also from *Alstonia venenata* and *Melodinus australis*. The first species produces also 5,22-dioxokopsan (**55**), the second one condylocarpine (**37**), both of which are secondary coalkaloids of strictosidine, and show further chemotaxonomic connections to two other special groups of alkaloids, i.e. **55** to the kopsan subgroup of the plumeran group, **37** to the aspidospermatan group. The seco alkaloid quebrachamine (**53**) isolated from *Rhazya stricta* is likewise an important key compound in the biogenesis of the type III alkaloids. In the eburnan group only two alkaloids were isolated from strictogenic species, one of them is dihydroeburnamenine (**62**, Scheme 12) (from *Rhazya stricta*) which is probably the last coalkaloid in this molecular evolution.

From these data it may be concluded that most of the characteristic groups of indole alkaloids have primary (direct) and/or secondary/tertiary (indirect) coalkaloids of strictosidine, and they form an extended biogenetic network. However, at certain points there are holes on this net. In the isoibogan and isoeburnan groups no coalkaloids of strictosidine were found, a fact whch can easily be interpreted by the low number of known isolated compounds in them. In the isoplumeran and aspidospermatan groups the absence of coalkaloids can hardly be attributed to any low statistical probability. However, this lack of coalkaloids is compensated by the high number of dimeric alkaloids having an intact or seco isoplumeran subunit (see below) and by a strong chemotaxonomic relation between the aspidospermatan and the strychnan groups.

At this point the importance of the two strictogenic species of the Apocynaceae family should be emphasized. As seen in Table 1, only a small number of coalkaloids were isolated from *Anthocephalus cadamba* of Rubiaceae and *Strychnos mellodora* of Loganiaceae having the malindan and vincosaman skeletons, respectively. However, in the Apocynaceae family from *Catharanthus roseus* (=Vinca rosea) 30 monomers and 20 homo- or heterodimers, from *Rhazya stricta* 50 monomers, 8 homodimers, as well as some stemmadenine and secodine derivatives of key importance were isolated. Most of them have the corynanthean, and only a few the vallesiachotaman skeleton. These two species together provide about 130 primary coalkaloids, of which only two are common in the two species!

**Scheme 10:**
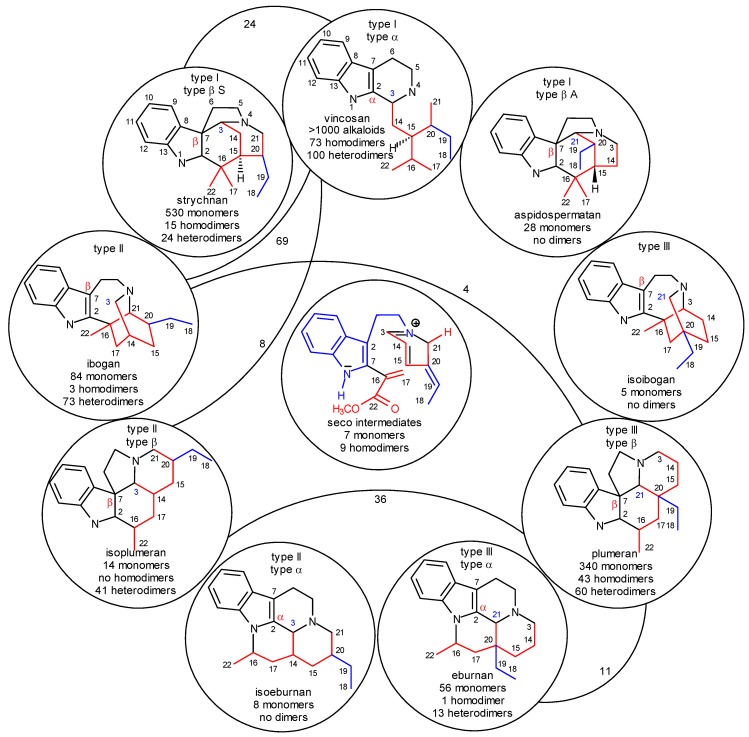
Types of Heterodimer Indole Alkaloids.

## The Significance of Dimer Alkaloids in the Biogenesis of Indole Alkaloids

In the class of the indole alkaloids nearly 300 dimer compounds are known, which are formed from two monomer units (each monomer unit contains a tryptamine and a secologanin subunit). In the minority of these dimers the two monomers or at least their basic skeletons are identical (“homodimers”). From chemotaxonomic point of view the dimers having two different basic skeletons (“heterodimers”) are much more important because they indicate that in the species from which they were isolated the different monomers coexist, i. e. their origin is common: they were formed directly one from the other or indirectly from common precursors. Therefore their biogenetic relatedness is evident.

It is not the aim of this paper to analyze the mechanism of the formation of these dimers and investigate the types of the molecular connection between the monomer units (this study is in progress). The types of the heterodimers and the numbers of the subunit monomers are shown in Scheme 10. The connection may concern either the tryptamine or the secologanin subunit, or both of them. In the group of the seco intermediates (stemmadenine, secodine and secamine derivatives in the center of Scheme 10) no heterodimers were found. The phenomen, perhaps, depends on the chemical character of their ring systems. Likewise no dimers having either an isoibogan or an isoeburnan monomer are known, as even the number of the known free monomers is low.

**Scheme 11:**
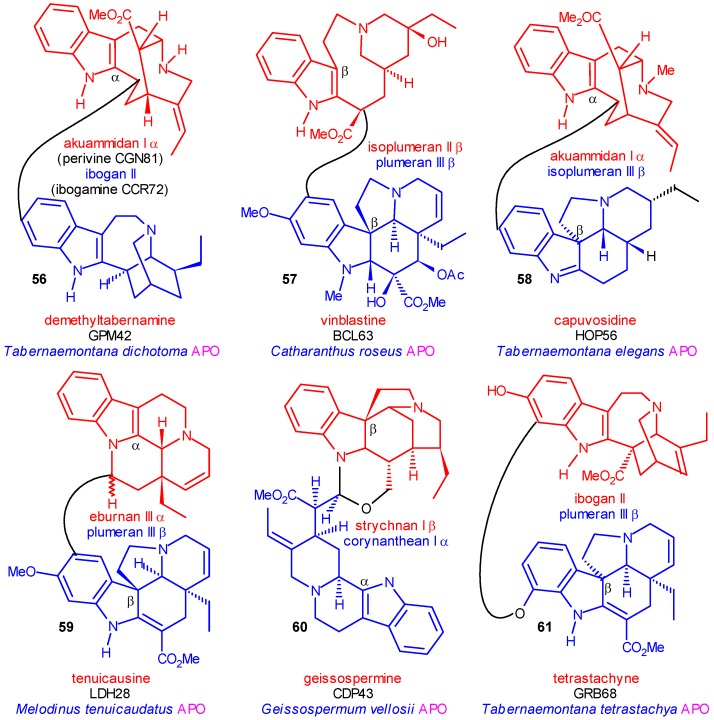
Selected Examples of Heterodimer Indole Alkaloids.

Some examples of existing heterodimers are shown in Scheme 11. In geissospermine (**60**) and tenuicausine (**59**) the monomer components belong to the same type of skeleton (both type I or type III, respectively), but their attachment to the indole ring is different (α and β). **60** suggests an α→β bond shift, **59** a backward β→α shift between the two monomers.

In tetrastachyne (**61**) and vinblastine (**57**) (with several derivatives) the monomers have two differently rearranged skeletons. It proves that type II and type III skeletons coexist in the cells, and should be formed at least in the same species. Several derivatives of vinblastine (**57**) emphasize the importance of this connection. Moreover, it was mentioned that no isoplumeran monomer is known as coalkaloid of strictosidine, however, in the **57** and its derivatives some isoplumeran subunits (in seco form) became important coalkaloids because most of the dimers of type **57** were isolated from the strictogenic *Catharanthus roseus* (=*Vinca rosea)*. In **56** and **58** one of the monomers has a (differently) rearranged skeleton (type II or type III), and the other monomer has the original secologanin skeleton. These facts suggest that the monomers having an intact skeleton and any of the rearranged ones are produced of the same species. In **58** one of the components is again an isoplumeran monomer (now in its intact form) and by this fact its significance is further increased. Finally, in demethyltabernamine (**56**) the two monomers are perivine (akuammidan- and mainly vobasan-type) and ibogamine (ibogan-type). The dimer and both monomers were isolated from several *Tabernaemontana* species and in addition perivine from the strictogenic *Vinca rosea* as well. This latter fact is very important because it represents the chemotaxonomic link between strictosidine and the tabernamine-type dimers (about 70 alkaloids!).

## Outlook

**Scheme 12:**
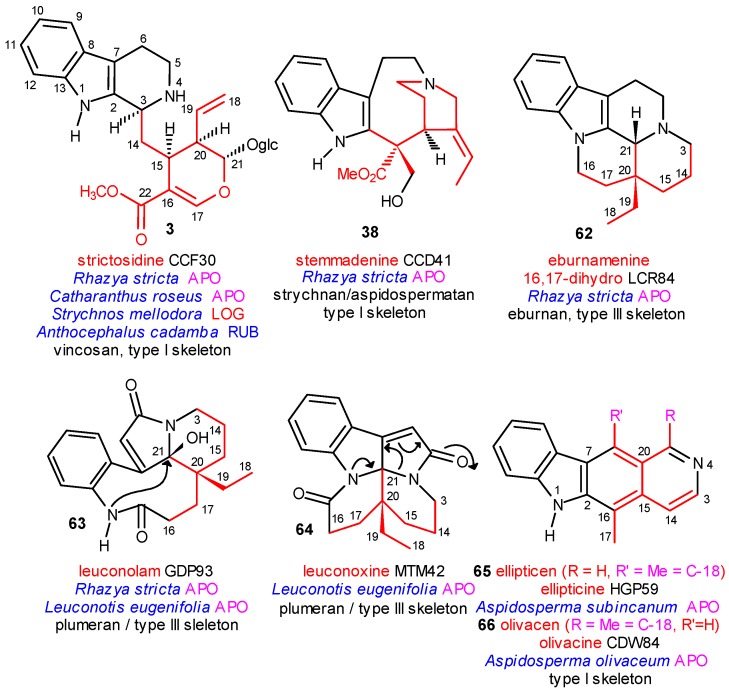
Some Special Structures.

The first line of Scheme 12 shows once more the precursor strictosidine 1, the central intermediate stemmadenine (**38**) and dihydroeburnamenine (**62**) as one of the final products of this long molecular evolution.

In the second line the unusual leuconolam (**63**) and leuconoxine (**64**) as well as the ellipticine–olivacine pair **65** and **66** are placed which look to be, at least in appearence, very different to strictosidine or even stemmadenine, nevertheless they could find their appropriate position in the system. Compound **63** was isolated from the strictogenic *Rhazya stricta* and in addition from *Leuconotis eugenifolia*, in which **64** was also found as a secondary coalkaloid of strictosidine.

**Scheme 13:**
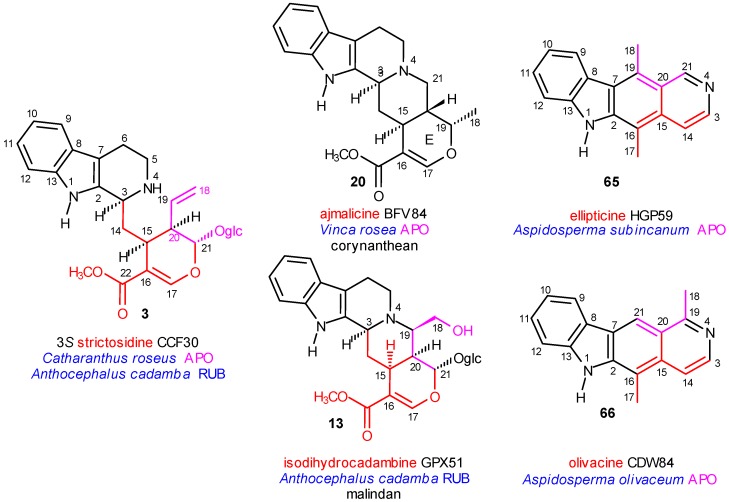
A Special Case.

In Scheme 13 the case of the ellipticine–olivacine pair illustrates well the potential of the electronic searching in the special data bases [14]. Both compounds **65** and **66** are type I class alkaloids and structural isomers as the position of C-18 is different in the common ringsystem. They were isolated from closely related *Aspidosperma* species (Apocynaceae). It could be demonstrated, that their evolutionary lines branched at the very beginning, on the primary azacyclization level.

The evolution of ellipticine (**65**) started from ajmalicine (**20**) (coryanthean skeleton with nearly thousand alkaloids) isolated from the strictogenic *Catharanthus roseus* (Apocynaceae) and the main steps of its evolutionary line could be followed stepwise [14]. In the other case only the first and last compounds were found as natural products. The evolution of olivacine (**66**) started from isodihydrocadambine (**13**) (malindan skeleton with 40 alkaloids) isolated from the likewise strictogenic *Anthocephalus cadamba* (Rubiaceae) but its formation could not be followed stepwise because of the low number of isolated alkaloids having the malindan skeleton. However, it may be stated with high probability that the molecular evolution of olivacine passed steps analogous to those of ellipticine.

## Summary

In this paper chemotaxonomic investigations were carried out on 133 monomer and 25 dimer coalkaloids, as well as on 152 dimers of strictosidine. The results are summerized in Table 2. It can be seen, that the isolated compounds form a biogenetic network. Coalkaloids and/or heterodimers were found in all three basic types I, II and III, and nearly all important taxonomic units of indole and quinoindole alkaloids (except such groups in which only a few alkaloids are known). Of course, the search for further indirect (secondary/tertiary) coalkaloids could and should be continued, in order to make the molecular network more dense. The results underline also the importance of the computer searching in the specialized large databases.

**Table 2:** Summary of Chemotaxonomic Relationships between Indole Alkaloids.